# A Rare Cause of a Fluctuating Cystic Lesion in the External Auditory Canal

**DOI:** 10.1155/2018/6197314

**Published:** 2018-01-23

**Authors:** Aaron Esmaili, Allen Y. Y. Wang, Jafri Kuthubutheen

**Affiliations:** ^1^Department of Otolaryngology, Head and Neck Surgery, Fiona Stanley Hospital, Perth, WA, Australia; ^2^Department of Otolaryngology, Head and Neck Surgery, Royal Perth Hospital, Perth, WA, Australia

## Abstract

Temporomandibular joint (TMJ) ganglionic cyst is an uncommon entity and only a few have been reported in the literature. TMJ ganglion within the external auditory canal presenting clinically as a fluctuating cystic lesion has never been reported. Here, we present a unique case of such a lesion together with otoscopic and radiological images as well as provide a descriptive review of TMJ ganglionic cysts.

## 1. Introduction

Ganglion cyst is expansile, synovial fluid-filled lesion of joints, most commonly seen in the wrists, knees, and feet. Reports of these cystic lesions arising from the temporomandibular joint (TMJ) are scarce. When they occur, they are most commonly found in the preauricular region, with the most common presenting clinical features being pain and swelling [1]. Conventionally, pain, limitation of movement, and local pressure symptoms are indications for surgery [2]. Due to the direct visibility of the preauricular region, aesthetics plays a part for opting toward surgical management even in the asymptomatic patient. In contrast, TMJ ganglion presenting as a lesion in the external auditory canal (EAC) is not usually visible, and therefore asymptomatic patients may not require treatment.

## 2. Case

A 59-year-old female was referred to a tertiary otorhinolaryngology unit for multiple reasons. Of these, the predominant troubling symptom was bilateral blocked ears, the right being worse than the left. This was associated with intermittent sharp retroauricular pain, a history of chronic tinnitus, and chronic headaches. She denied history of otalgia, otorrhoea, vertigo, hearing loss, TMJ pain, and discomfort even in the setting of mastication.

**Figure 1:**
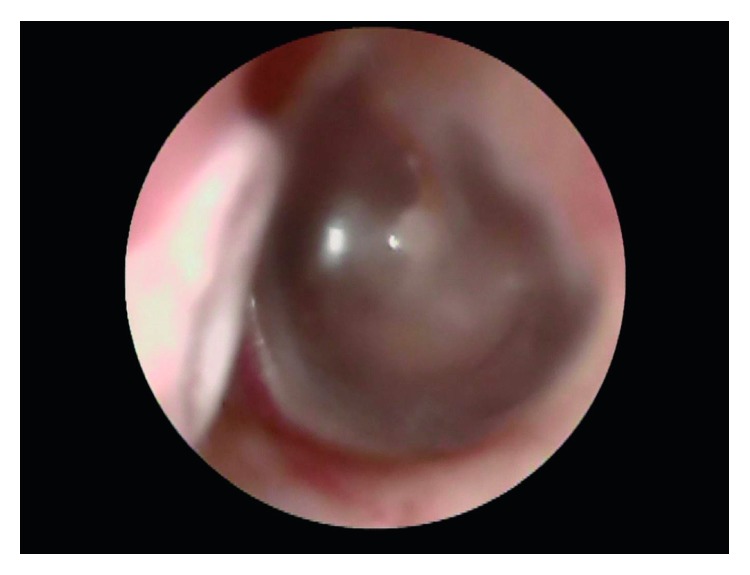
Otoscopy of the left external auditory canal. TMJ ganglion cyst (deflated) seen in the anterior inferior wall of the left external auditory canal.

On examination, there was absence of TMJ crepitus. The cranial nerves were intact, and in particular there was no facial nerve weakness or facial paraesthesia. Otoscopy revealed a cystic lesion arising from the anterior inferior wall of the left external auditory canal (Figure 1). The cystic lesion fluctuated in size with opening and closing the jaw (available here). Pure tone audiogram revealed hearing thresholds within normal limits bilaterally.

**Figure 2:**
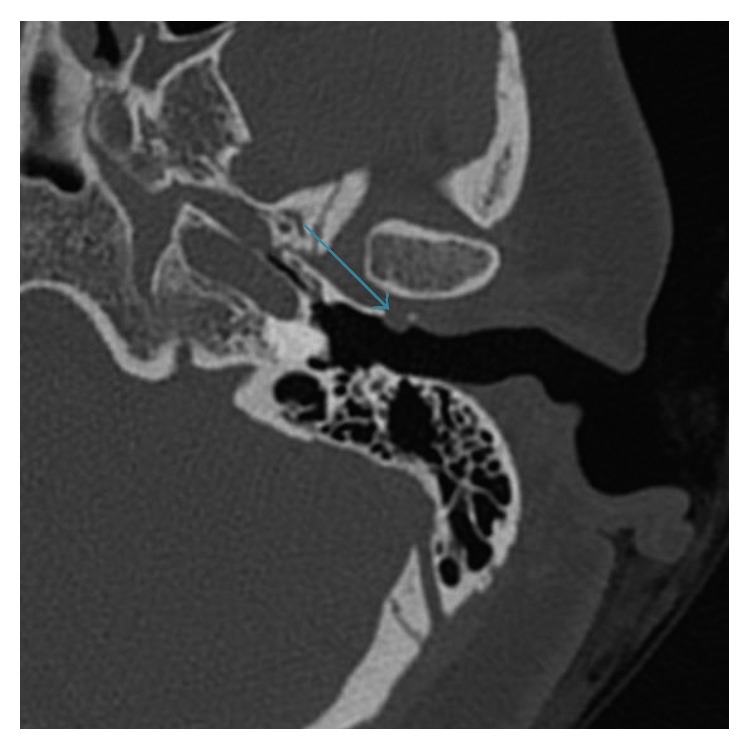
Axial noncontrast computed tomography of the temporal bone. A TMJ ganglion cyst is noted.

A presumptive diagnosis of TMJ ganglion was made. Computed tomography revealed that the cystic lesion communicated with the TMJ (Figure 2). At follow-up surveillance, there was no progression of the cystic lesion, and given the patient was asymptomatic of the lesion, a decision was made not to proceed with surgical excision.

## 3. Discussion

Ganglion cyst is a benign soft tissue lesion that usually is found near joints most notable wrist, hand, and feet. TMJ ganglionic cyst is an uncommon entity, and only a few have been reported in the literature. TMJ ganglion within the external auditory canal is rare. The median age of patients is approximately 46, and TMJ cysts occur more commonly in the female gender with a ratio of 3 : 1 [1].

The aetiology of ganglionic cysts remains largely unknown. They are thought to come about from myxoid degeneration of the connective tissue encompassing the joint capsule. Trauma and inflammation have been suggested to play a role [1, 3].

The most common symptoms are cystic swelling and pain. Various other symptoms have been reported including neuropathic pain, pain on mastication, pain on awakening, trismus, paraesthesia, TMJ crepitus, and facial nerve weakness [1]. Preauricular region appears to be the most commonly affected location with swelling [3]. Within the literature, including this case, there are only four reported cases of TMJ ganglion presenting as an external auditory canal (EAC) mass [4–6]. Symptoms varied and included frequent otitis externa, aural fullness, hearing loss, and otorrhoea. Otalgia and typical TMJ syndrome symptoms were not a feature in these patients. The most common cause of mass within with EAC is exostosis and osteomas [5]. In patients where the appearance of the EAC lesion is atypical, imaging is often indicated. In all the previous documented cases [4–6], imaging was utilised. Computed tomography with or without MRI appears to be useful diagnostic tools. Arthrography may also be considered in making the diagnosis. From a histopathological point of view, ganglion cysts are lined by fibrous tissue having arisen from myxoid degeneration of connective tissue from joint capsule [3].

As with ganglion elsewhere surgery is a mainstay form of management. However, if diagnosis can be made confidently based on clinical history, examination, and imaging in an asymptomatic patient, a period of surveillance is justified. However, if the patient is symptomatic, surgery with complete resection appears to provide relief of symptoms [6]. The patient in the present case was largely unaffected by her TMJ cyst and demonstrated normal hearing thresholds; therefore, surveillance was prescribed. On close clinical follow-up, the patient remained asymptomatic of the lesion, and there was no progression.

## 4. Conclusion

In summary, we provide the first report of a TMJ ganglion presenting as a fluctuating cystic lesion within the EAC. This patient did not have any TMJ pain or otalgia. Importantly, the cystic lesion remained stable. Imaging especially CT provides guidance in making the diagnosis. Surgical excision is an option in symptomatic patients; otherwise, conservative management remains a good choice.

## Supplementary Materials

Supplementary Materials The otoscopic view of the fluctuating cyst within the external auditory canal. The patient is asked to open and close her mouth resulting in fluctuation of the cyst.
